# Comparison of Choroidal Thickness Measurements Using Spectral Domain Optical Coherence Tomography in Six Different Settings and With Customized Automated Segmentation Software

**DOI:** 10.1167/tvst.8.3.5

**Published:** 2019-05-02

**Authors:** Helena Giannakaki-Zimmermann, Wolfgang Huf, Karen B. Schaal, Kaspar Schürch, Chantal Dysli, Muriel Dysli, Anita Zenger, Lala Ceklic, Carlos Ciller, Stephanos Apostolopoulos, Sandro De Zanet, Raphael Sznitman, Andreas Ebneter, Martin S. Zinkernagel, Sebastian Wolf, Marion R. Munk, on behalf of the Bern Photographic Reading Center

**Affiliations:** 1 Department of Ophthalmology and Department of Clinical Research, Inselspital, Bern University Hospital, and University of Bern, Switzerland; 2 Bern Photographic Reading Center, University Hospital Bern, Switzerland; 3 Karl Landsteiner Institute for Clinical Risk Management, Vienna, Austria; 4 RetinAI Medical AG, Bern, Switzerland; 5 ARTORG Center for Biomedical Engineering Research, University Bern, Bern Switzerland; 6 Department of Ophthalmology, Northwestern University, Feinberg School of Medicine, Chicago, Illinois, USA

**Keywords:** choroicalpillaris, OCT, functional imaging

## Abstract

**Purpose:**

We investigate which spectral domain-optical coherence tomography (SD-OCT) setting is superior when measuring subfoveal choroidal thickness (CT) and compared results to an automated segmentation software.

**Methods:**

Thirty patients underwent enhanced depth imaging (EDI)-OCT. B-scans were extracted in six different settings (W+N = white background/normal contrast 9; W+H = white background/maximum contrast 16; B+N = black background/normal contrast 12; B+H = black background/maximum contrast 16; C+N = Color-encoded image on black background at predefined contrast of 9, and C+H = Color-encoded image on black background at high/maximal contrast of 16), resulting in 180 images. Subfoveal CT was manually measured by nine graders and by automated segmentation software. Intraclass correlation (ICC) was assessed.

**Results:**

ICC was higher in normal than in high contrast images, and better for achromatic black than for white background images. Achromatic images were better than color images. Highest ICC was achieved in B+N (ICC = 0.64), followed by B+H (ICC = 0.54), W+N, and W+H (ICC = 0.5 each). Weakest ICC was obtained with Spectral-color (ICC = 0.47). Mean manual CT versus mean computer estimated CT showed a correlation of *r* = 0.6 (*P* = 0.001).

**Conclusion:**

Black background with white image at normal contrast (B+N) seems the best setting to manually assess subfoveal CT. Automated assessment of CT seems to be a reliable tool for CT assessment.

**Translational Relevance:**

To define optimized OCT analysis settings to improve the evaluation of in vivo imaging.

## Introduction

The choroid, being a connective tissue, contains a vascular meshwork and is essential for adequate retinal function as it supplies nutrients and oxygen to the retinal pigment epithelial cells and photoreceptors.^1^,^2^ With introduction of optical coherence tomography (OCT) into the clinical routine and especially with the capability of using “enhanced depth imaging” (EDI) and swept source OCT (SS-OCT), the choroid can be visualized in more detail, which now allows evaluation of the choroid in healthy and in diseased eyes.^1^,^3^ In several diseases, such as central serous chorioretinopathy, Vogt Koyanagi Harada disease, polypoidal choroidal vasculopathy, and age-related macular degeneration, evaluation of the choroid and CT has become a valuable additional tool to follow disease activity and progression and consequently are subjects of investigation.^4^–^8^

When evaluating subfoveal CT manually, prior studies found a difference in CT in the human choroid, ranging from 220 to 300 μm at the posterior pole, and from 100 to 150 μm at the periphery in healthy eyes.^9^ These differences contributed to a lot of factors, such as age,^10^ refractive error,^11^ daytime, smoking, sex, and hemodialysis.^12^ In pachychoroid disorders, including central serous chorioretinopathy, the CT is increased compared to normal eyes.^13^

The inconsistency of vessel diameters at the posterior pole, with a higher concentration of arteries and different-sized choroidal veins, may result in a variability of CT measurements.^14^,^15^ In some individuals the suprachoroidal layer corresponds to a hyporeflective band, which may further alter the CT measurements and complicate the precise measurement.^16^ Despite the challenges of manual CT measurements, the reproducibility has been high with strong inter- and intragrader correlations.^17^ Interdevice agreement among different spectral domain (SD)-OCT devices and between SS-OCT and SD-OCT may slightly vary, but seems to be overall strong.^18^,^19^ Beside manual assessment, CT also may be evaluated using automated segmentation and respective automated algorithms were described to be precise as well.^20^,^21^

Previously, we were already able to demonstrate that changing the contrast and color settings using the inbuilt OCT software may facilitate the differentiation of retinal structures and enable a better identification of retinal pathologies.^22^ However, it remains unclear which OCT settings are “the best” to accurately outline choroidal borders and to determine CT. Therefore, the primary purpose of this study was to identify the most reliable OCT setting to determine CT, and whether those measurements are consistent among different graders, within the same grader, and how they differ from an automated segmentation software.

## Materials and Methods

A total of 30 eyes of 30 Caucasian subjects were included in this study. Consecutive EDI-OCT images were selected retrospectively from patients who underwent an EDI-OCT examination as part of their ophthalmologic appointment. Table 1 describes the patients/pathologies. All patients were imaged using SD-OCT and the horizontal central line scan of the posterior pole was used for evaluation. All participants had clear media and were scanned in miosis. Ethics approval (KEK-Nr. 093/13) to conduct this study was obtained from the local ethics committee, and the study was performed in accordance with International Conference on Harmonisation-Good Clinical Practice (ICH-GCP) guidelines. Due to the retrospective design of the study, waiver of written informed consent was granted.

**Table 1:** Incidence of Retinal Disorders in the Cohort Group

N = Absolute Number	Retinal Disorders
11	No retinal disorder – normal
6	Diabetic macular edema (DME)
6	Age-related macular degeneration (AMD)
2	Adult foveomacular vitelliform dystrophy
3	Epiretinal membrane
1	Central vein occlusion
1	High myopia with choroidal neovascularization (CNV) and retinoschisis

### OCT Imaging

All patients were scanned using EDI in 840 nm SD-OCT (Software version 5.3; Spectralis SD-OCT; Heidelberg Engineering, Heidelberg, Germany). Scans were acquired using an established protocol of a single horizontal line scan. Images were acquired in the high speed mode, had a scanning angle of 30° and consisted of 36 frames per B-scan using the average real time mode (ART). Only scans through the fovea and images with a high signal-to-noise ratio (in dB) with a minimum of 20 dB were included and used for evaluation of the CT.

### Image Processing

All 30 EDI-OCTs were exported from the Heidelberg software and saved in TIFF format using six different predefined settings (three different color settings and two different contrast settings), resulting in a total of 180 images.

The settings were: positives and negatives—as referred to in the terminology of black and white photography and Spectrum color encoded images used by the Heidelberg software system. Both achromatic settings show an inverted gray scale. Positive images showing a black image on a white background, negative images showing a white image on a black background.^22^ The Spectrum color scale uses a rainbow spectrum in which the maximal signal down to no signal is represented by white-red-orange-yellow-green-blue-black, and each pixel value is assigned to a color. The images were extracted at the Heidelberg predefined normal contrast setting (contrast scale 9 in Heidelberg software for black-on-white images and Spectrum color-coded images, and contrast scale 12 for white-on-black images) and in high contrast (contrast scale 16 in Heidelberg software), finally resulting in the following images per EDI-OCT: white/normal (W+N) = white background with black image at normal contrast 9, white/high (W+H) = white background with black image at maximal contrast of 16, color/normal (C+N) = Spectrum color-encoded image on black background at predefined contrast of 9, color/high (C+H) = Spectrum color-encoded image on black background at maximal contrast of 16, black/normal (B+N) = black background with white image at predefined contrast of 12, and black/high (B+H) = black background with white image at maximal contrast of 16.

**Figure 1:**
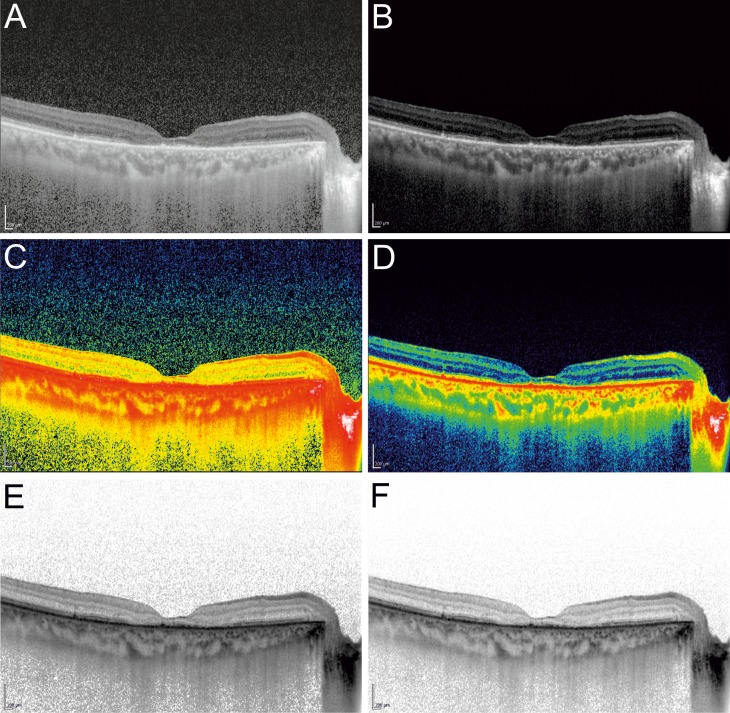
Six images depicting the different settings of each EDI-OCT scan. (A) B+H. (B) B+N. (C) C+H. (D) C+N. (E) W+H. (F) W+N.

The different settings are shown in Figure 1.

### Manual Grading of Subfoveal CT

Nine experienced graders from the Bern Photographic Reading Center (BPRC) and a customized automated segmentation software from the ARTORG Center for Biomedical Engineering Research Bern evaluated the CT. The scleral-choroidal border was defined as the zone of variable reflectivity at the outer border of the choroidal vasculature. In case of the presence of a suprachoroidal, hyporeflective band, a respective band was included in the measurement.^16^ Before assessment, the graders were trained to measure the subfoveal CT as the distance between the posterior edge of the retinal pigment epithelium (RPE)-Bruch's membrane complex and the choroidal–scleral interface.

The 180 TIFF images were randomly ordered, and each grader went through the dataset in the same chronology. All images were viewed using a harmonized screen setting in a standardized dimmed environment.

Independent, masked measurements were done using ImageJ software (IJ 1.46r).^23^ Scaling factor was determined beforehand by one experienced grader (MRM) and the same was applied for all images and all graders. Absolute measured values of the subfoveal CT were taken down in an individual table for each grader made with SPSS (Released 2013, IBM SPSS Statistics for Windows, Version 22.0; IBM Corp., Armonk, NY). Beside the quantitative measurements the graders also were asked to subjectively assess distinguishability of the choroidal border, grading the image with a number between 1 (poor depiction of the choroidal border) and 10 (excellent and perfect depiction of the choroidal border).

### Automated Algorithm for Assessment of Subfoveal CT

The automated algorithm to detect the choroidoscleral border and assess subfoveal CT was initially trained on a dataset using individual EDI B-scans with manually delineated sclero-choroidal borders.

The segmentation of the choroidal surface was based on a modified version of the deep learning approach proposed by Apostolopoulos et al.^24^ In this approach, a Convolutional Neural Network (CNN) was used to segment the choroidal surface (foreground) from the rest of the retina (background). Given an EDI-OCT image as input, the CNN outputs a probability map of the same dimensions, with a range of 0 to 1, where 0 was defined as the background and 1 was defined as the foreground. The operating point for the segmentation was set to 0.5, that is, pixels with a probability equal or higher than 0.5 were considered part of the choroid.

**Figure 2:**
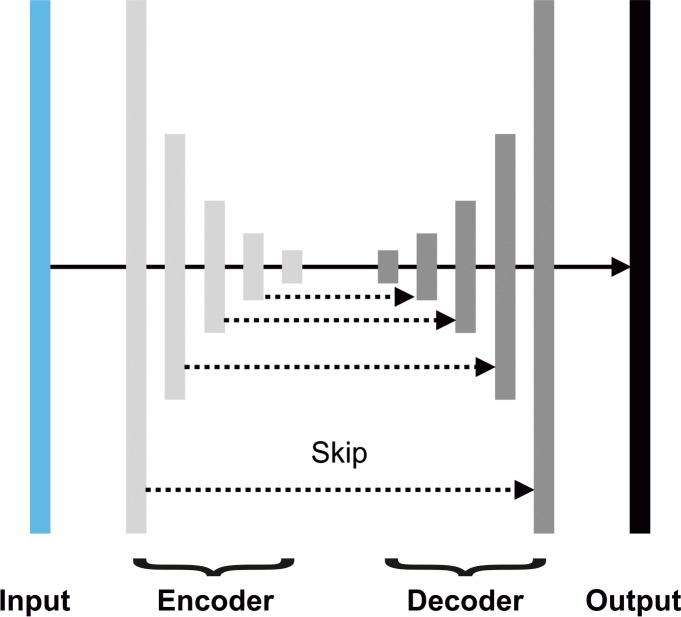
Encoder-decoder network configuration for automatic segmentation of the choroidal surface. The EDI B-scan is processed a set of encoder and decoder layers, each consisting of convolutional blocks followed by a contracting (encoder) or expanding operation (decoder). The skip connections connect corresponding layers to improve the flow of information through the network. The output is a probability map of the same size as the input image.

The CNN was designed as an encoder-decoder configuration, where each input image is processed by a series of convolutional blocks and contracting operations (encoder layers), followed by a series of convolutional blocks and expanding operations (decoder layers; Fig. 2). Contracting operations reduce the resolution of the image by a factor of 2, while expanding operations increase it by the same factor. Each convolutional block contains three sets of 1 × 1, 3 × 3 and 5 × 5 convolution kernels, which are learned through an optimization process. Skip connections link corresponding encoder and decoder layers to improve the flow of information, while a side-branch connects the input image with every encoder layer to provide context at multiple scales. Hyperparameters were set according to Apostolopoulos et al.^24^

The network was optimized using backpropagation to minimize the difference between the predicted choroidal surface and a manual ground-truth annotation from an expert grader. The loss function was defined as the weighted sum of the binary cross-entropy between the predicted and the true choroidal surface (weight 0.9), and the total variation of the predicted surface (weight 0.1). The optimization process relied on Stochastic Gradient Descent (SGD) with an initial learning rate of 10^−3^, which was halved every 20 iterations, and continued until the loss function converged.

The network was trained on 148 manually annotated EDI-OCT images of 40 subjects, which were mirrored horizontally (296 images) and then augmented with random elastic deformations. There was no overlap between the training and testing sets of EDI-OCT scans.

After convergence, the algorithm was applied to the test set. Its output was compared to the results of the graders on the same images.

### Statistical Analysis

Data was collected using SPSS 21 (SPSS, Inc., Chicago, IL) and statistical analysis was performed using the free software R (available in the public domain at www.R-project.org). Based on the previous study of Palma et al.,^22^ we assumed that the level of the ICC of the CT measurements reflects best the superiority of a setting. Thus, we assumed that the higher the ICC the more reliable was the OCT setting to determine CT. Therefore, quantitative data were analyzed using the intraclass correlation (ICC) coefficient. Fleiss κ was applied to evaluate the agreement in terms of distinguishability of the choroidal border. Pearson correlation coefficient was used to assess the correlation of subfoveal CT between mean manual grading and automated segmentation. To identify a potential significant difference among the individual ICCs of the different settings, a bootstrap analysis was used to compare ICC coefficients. For all analyses, *P* < 0.05 was considered statistically significant. The procedure (false detection rate, FDR) described by Benjamini and Hochberg^25^ was used to adjust *P* values for multiple testing. Absolute values are stated in mean values and their standard deviations.

## Results

**Figure 3:**
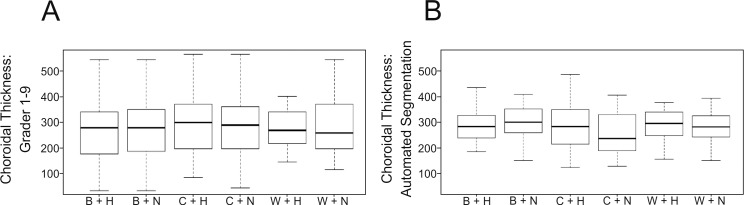
(A) illustrates the 95% confidence interval (CI) and mean CT in respect to each individual setting (B+H, B+N, C+H, C+N, W+H, W+N). (B) Depicting the 95% CI and mean values of the customized segmentation software with respect to each setting.

Mean values with respect to each setting of each grader and of the automated segmentation can be found in Supplementary Figure S1. Mean CT values with respect to each setting are outlined in Figures 3A and 3B.

### Intergrader Consistency: Means of CT and Heteroscedasticity

**Figure 4:**
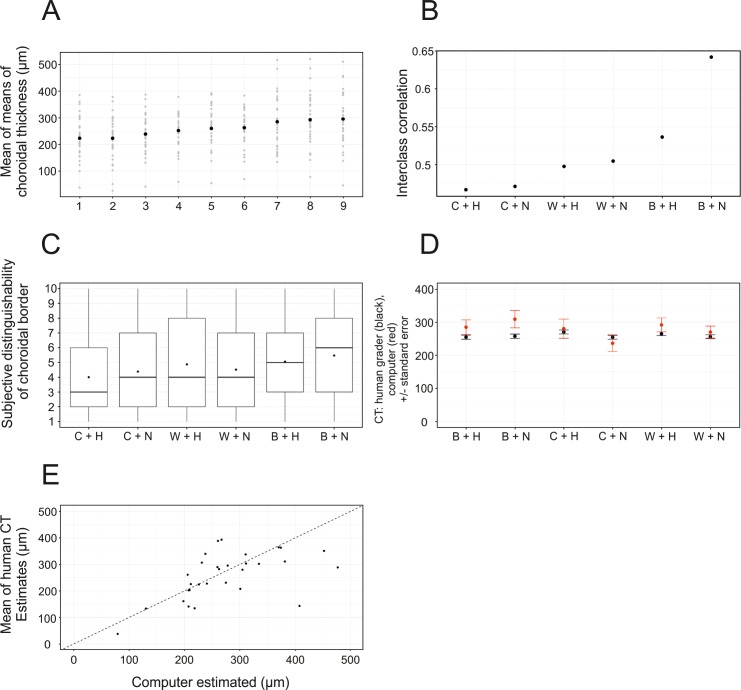
(A) Mean CT of each grader (1–9) irrespective of predefined setting. The dark black dot illustrates the mean, while the gray dots represent the range of CT measurements. (B) ICC showing the correlation of all graders depending on the setting. Correlation was highest for the B+N setting and lowest for the C+H setting. (C) Subjective distinguishability of choroidal boarder, rating from 1 (poor distinguishability) to 10 (perfect distinguishability) of all graders with respect to each setting. Subjective distinguishability was highest for the B+N setting with an average grade of 6. (D) Comparison of human versus computed absolute measurements (um) of the CT, each column representing the different settings. Human results are shown in black, computed results in red. On average, computed CT was estimated thicker than the CT measured by the human graders. (E) Correlation between mean CT measured by the human graders versus mean computer-estimated CT (Pearson correlation r = 0.6, P = 0.001).

To assess the agreement of CT measurements among the graders, the overall means of CT measurements including the measurements of all predefined settings were evaluated. Mean CT values of each grader can be found in Figure 4A. There was a maximal difference of 70 μm among the graders: Grader 1 measured an overall mean CT of 223 ± 83 μm, whereas grader 9 measured in mean 295 ± 103 μm, independently of which setting was selected (Fig. 4A). Interestingly, the variability and differences among the measurements within the individual graders were greater the thicker the mean estimated CT was (Supplementary Fig. S2).

### Intergrader Correlation in Respect to Different Settings

To assess which of the six predefined settings was most suitable for evaluation of subfoveal CT and, thus, which setting shows the highest agreement among the graders, ICC correlation was used. In general, the ICC was higher in normal than in high contrast images, better for achromatic black than for white background images, and achromatic white background images were better than the color images (Fig. 4B). Accordingly, the highest ICC was achieved in the black/normal setting with an ICC coefficient of 0.64, followed by the black/high setting (ICC coefficient = 0.54) and the white/high and white normal settings (ICC coefficient = 0.5 each; Fig. 4B). The weakest results were achieved in the color settings with an ICC coefficient of 0.47 in the color/normal and color high settings, respectively (Fig. 4B).

**Figure 5:**
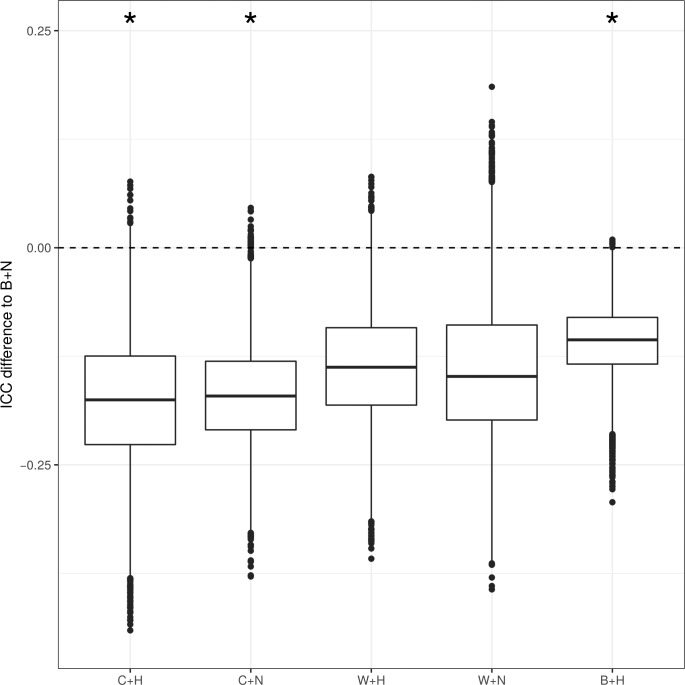
Bootstrap analysis illustrating the difference of the ICC of each setting compared to the B+N setting. Considering the ICC of B+N as reference value (in the Figure illustrated with the dashed line at 0), the distributions of the differences to the ICCs of the other settings are plotted. After P value adjustment using false detection rate, a procedure described by Benjamini and Hochberg,^25^ the ICCs of B+N remained significantly higher than the B+H, C+H, and C+N settings. The ICCs of the CT measurements using a white background (W+H and W+N) were not significantly different after P value correction.

B+N achieved the highest ICC and, therefore, was chosen to be tested for its potential to be superior to the remaining settings. Indeed, bootstrap analysis (using 10,000 samples) revealed that the B+N setting achieved significantly higher ICCs and was superior to all other settings (Fig. 5). After *P* value correction using FDR, the B+N setting remained superior compared to the B+H (*P* = 0.0005, corrected *P* = 0.015), C+H (*P* = 0.0044, corrected *P* = 0.044), and C+N (*P* = 0.003, corrected *P* = 0.044) settings. W+N (*P* = 0.049, corrected *P* = 0.29) and W+H (*P* = 0.018, corrected *P* = 0.13) did not remain statistically significant after *P* value adjustment.

### Subjective Distinguishability of Choroidal Border

In keeping with the ICC coefficient, best subjective distinguishability of choroidal border was awarded to images with B+N (black background and normal contrast), with a mean grade of 5.5 (median 6), followed by the B+H (black/high) setting (mean 5.1, median 5; Fig. 4C). Accordingly, the W+H (white/high) and W+N (white/normal) settings were mid-table with a mean of 4.9 (median 4) and4.5 (median 4), respectively. The choroidal border was poorest detectable on the predefined color settings (C+N, mean = 4.4, median 4; and C+H, mean = 4.0, median = 3, respectively; Fig. 4C).

### Human Grading Compared to Computed Grading

In 10 images, the algorithm failed to delineate the sclero-choroidal border: The majority of segmentation failures was seen in the color/high setting (six images), followed by the color/normal setting (three images), and one provided in the white/normal setting. The pooled mean subfoveal CT of the manual graders and mean subfoveal CT of the computer estimated CT in respect to different predefined settings can be seen in Figure 4D. Mean CT of the manual human grading versus mean computer estimated CT showed a correlation of *r* = 0.6 (Pearson correlation, *P* = 0.001; Fig. 4E).

## Discussion

We evaluated the reliability of manual and automated CT measurements with the help of experienced graders and an automated algorithm. One main interest was whether changing the background color and contrast of foveal OCT single scans helped increase the accuracy of subfoveal CT measurements in a variety of macular diseases. Our results revealed the best intergrader consistency when measuring subfoveal CT using images with a black background and normal contrast enhancement. A similar correlation was found between the computer-estimated CT and manual measurements.

Despite this reasonable agreement, a significant discrepancy up to 70 μm was found among the graders when evaluating the overall mean CT values; a difference that is for sure clinically significant, in particular when CT is used to follow disease activity. Interestingly the variability and discrepancy among the graders was higher the thicker the mean estimated CT was. This mean intergrader difference should be considered, when CT is measured in clinical routine, used as an outcome parameter in clinical trials, and particularly when it serves as a follow-up parameter for disease activity. Thus, it would be beneficial if quantitative follow-up measurements were performed by one singular grader/clinician alone, to avoid falsely high or low CT values, which may impact assessment of disease progression or activity. This should be specifically considered in pachychoroidal diseases, central serous chorioretinopathy, anterior and posterior scleritis, and Vogt-Koyanagi-Harada disease.^13^ The thick choroid in respective diseases may lead to a high variability among the individual graders and measurements. Of note, this explicit difference was found even though all graders were well and homogeneously trained, graded CT according to a predetermined definition, and had the same environmental conditions, illumination, and screen resolution. Differences in training, screen settings, and illumination may even further increase the variability in CT measurements.

In a study in 2017, CT was analyzed to assess the distribution of CT on OCT in and outside of the vascular arcade, as well as in the foveal center in healthy eyes.^1^ CT was measured by two experienced graders. The ICC between these two observers was 0.876 (*P* < 0.05).^1^ Compared to our study, this ICC coefficient is much higher. Another study evaluated the CT of healthy eyes and revealed even higher ICC ranging from 0.91 to 0.98.^19^ The assessment of CT may, of course, be less challenging in healthy compared to diseased eyes, but also in further studies that focused on the CT measurement in diseased eyes, such as hydroxychloroquine retinopathy, central serous chorioretinopathy, or mitogen**-**activated protein kinase (MEK) inhibitor–associated retinopathy; they found an ICC ranging from 0.87 to 0.98 and a Pearson correlation of 0.97, respectively.^26^,^27^ Whether our results represented a normal range of the ICC when several graders (in contrast to only two graders) are analyzing CT remains unclear. All previously mentioned studies analyzed agreement between two graders, whereas to our knowledge our study was the first to explore the grading consistency of nine different graders, which might explain the higher variability of measurements. Our results highlighted that the accuracy of CT measurements may just not be as consistent and reliable as initially assumed from correlation of two graders.

One main research question was whether a certain setting may be able to facilitate CT measurement and lead to more accurate grading results. Our results clearly indicated that negative images with a black background and normal contrast offer the best conditions to allow accurate and correct CT grading results. Our findings also highlighted that the color-encoded settings are the most unsuitable to evaluate subfoveal CT. Whether correlations were best for the negative images with a black background, because graders might have been more familiar with this setting, remains speculative at this time point and also may be hard to evaluate in the future. A previous study from our group evaluated the impact of image contrast and color setting on the assessment of retinal structures on OCT. Interestingly, the background color and contrast settings the grader was accustomed to before barely affected the image assessment.^22^ In this study, we were not able to completely rule out this potential bias, because the all graders were used to the same setting, black background and normal contrast due to their clinical and grading routine.

In our study, color-encoded OCT images had the lowest intergrader consistency when delineating subfoveal CT. This is in line with a previous study of 2009 by Brar et al.^28^ who found that gray scale OCT images are qualitatively superior to color scale images for identification of different retinal features. Also, another study performed on the Stratus OCT demonstrated that gray scale images may have the potential to delineate additional information, previously not appreciable on the color-coded images.^29^ OCT images use a gray scale or a false color-coded scale based on reflected signal strengths. The standard inverted gray scale runs continuously from maximal signal (white) to no signal (black), while the false color scale uses a rainbow spectrum in which the maximal signal down to no signal is represented by white-red-orange-yellow-green-blue-black and each pixel value is assigned to a color.^22^,^28^ The investigators of the two prior studies believed that the pseudo-color images are presented in fewer tones, which leads to a grouping of different signal intensities.^28^ They further assumed that the transition of one color to another appears as a large difference to the human eye, but may represent only a subtle change in the underlying gray scale and pixel value.^28^ Our study was able to confirm prior assumptions, and it seems that these presumptions not only apply for the qualitative evaluation of retinal features, but also for the evaluation of choroidal structures.

Interestingly, respective difficulties using a pseudo-color–coded scale were not only seen by human graders, but also by the automated algorithm. In nine color coded images (in contrast to one image with a white background and in none with a black background) the algorithm failed to delineate the choroidoscleral border correctly. This confirms prior suggestions that image information may get lost when the image is depicted in false spectrum colors.

Agreement among graders and the computed-estimated CT measurements was good. Actually, the ICC of the CT between the human graders and the computed estimates was similar to the highest achieved agreement among the human graders; thus, similar to the ICC using the black/normal setting. This also is reflected when comparing the largest mean difference of CT measurements among the graders (70 μm) versus the largest disparity between mean CT of overall graders versus computer-estimated CT (52 μm, see Fig. 4D). In fact, there was less mean difference between the automated CT measurements and the human graders than among the individual human graders, which makes the automated algorithm an interesting and reliable tool to evaluate CT. Of course, more data are warranted to train the algorithm properly and to improve performance on a more varied set of disease patterns and imaging devices, but even with this limited number of available training data, the algorithm showed promising results. Additionally, the automatic algorithm is able to process two EDI B-scans per second, making it suitable for large-scale studies.

Our study has some limitations. As already mentioned, the graders were used mainly to validate black/normal images, which may have impacted respective results. Also, there is no gold standard to measure CT and to compare our CT measurements with. The “true” subfoveal CT remains unknown, even if there is a clear consensus from the graders. Variability of results also may vary depending on underlying retinal diseases and CT.

We have included eyes with various pathologies to determine a potential superior setting for the assessment of CT in general. Therefore, potential limitations include the fact that we did not evaluate the impact of different settings on CT measurements in particular macular pathologies. Therefore, we are (yet) unable to provide recommendations for specific eye conditions, such as intra- or subretinal hemorrhage but this might be assessed in future studies. Although the automated algorithm has a great potential to become a valuable tool in daily clinic use, it was so far only trained on a limited number of OCTs. Further studies using larger training datasets are warranted to improve its performance.

We demonstrated that color and contrast setting impact not only the evaluation of retinal structures as previously shown by Palma et al.,^22^ but also impact the accuracy of our quantitative CT measurements. Therefore, when analyzing CT, appropriate setting should be chosen and whenever possible a single grader/clinician should be selected to follow CT to reliably assess disease activity and progression.

In summary we can, nevertheless, state that a black background with white images at normal contrast (B+N) seems to be the best setting to manually assess subfoveal choroidal thickness with a reasonable consistency among the graders. Automated assessment of CT may also provide a reliable tool to evaluate CT.

## Supplementary Material

Supplement 1

Supplement 2
