# Supplementary material for: Comparison of Choroidal Thickness Measurements Using Spectral Domain Optical Coherence Tomography in Six Different Settings and With Customized Automated Segmentation Software
Source: Transl Vis Sci Technol. 2019 May 2;8(3):5. doi: 10.1167/tvst.8.3.5 (PMC6503890; doi:10.1167/tvst.8.3.5)
Supplement: Supplement 1 [file tvst-08-02-25_s01.pdf]

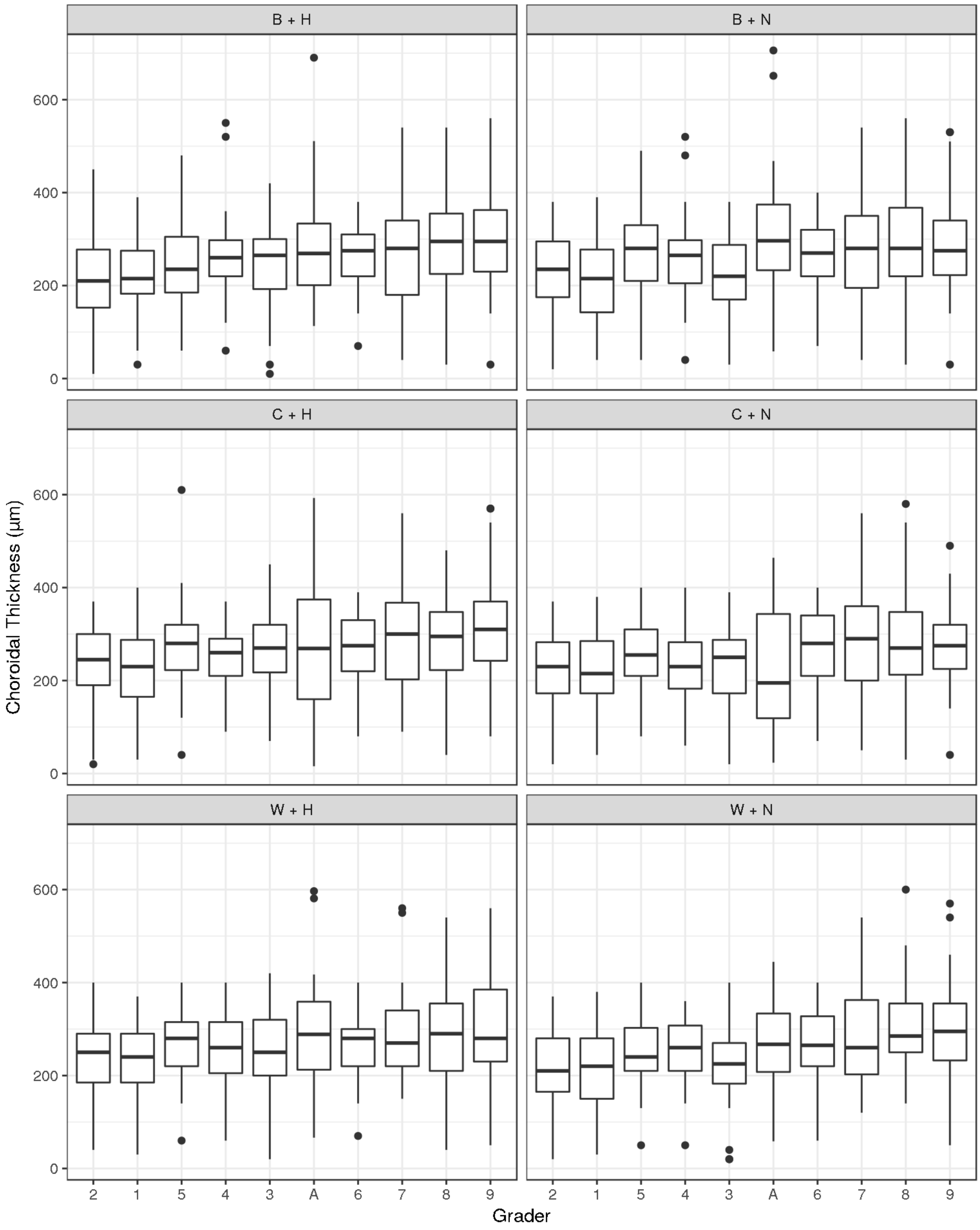

Supplemental Figure 1

Illustrates the mean values with respect to each setting of each grader (1-9) and of the automated segmentation (A).
