# Supplementary material for: Comparison of Choroidal Thickness Measurements Using Spectral Domain Optical Coherence Tomography in Six Different Settings and With Customized Automated Segmentation Software
Source: Transl Vis Sci Technol. 2019 May 2;8(3):5. doi: 10.1167/tvst.8.3.5 (PMC6503890; doi:10.1167/tvst.8.3.5)
Supplement: Supplement 2 [file tvst-08-02-25_s02.pdf]

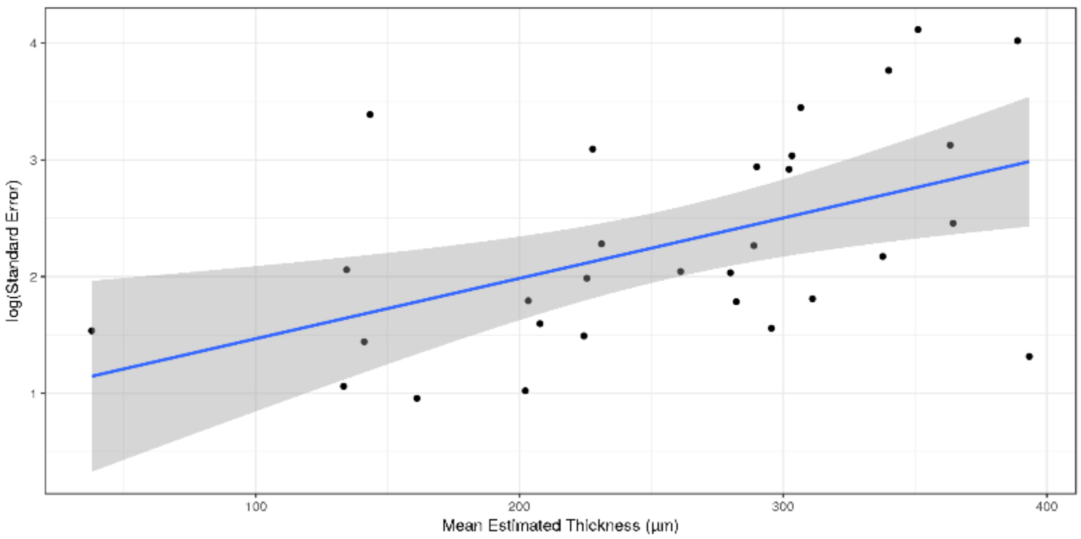


**Supplemental Figure 2**

Outlines the heteroskedasticity of the mean estimated choroidal thickness (CT) in the black and normal setting. The variability and discrepancy of the choroidal thickness measurements among the individual graders increased with increasing CT.
